# Longitudinal changes in infant non-nutritive sucking across the first year of life

**DOI:** 10.1016/j.earlhumdev.2025.106440

**Published:** 2025-11-24

**Authors:** Alaina Martens, Natalie Peterman, Kristen Allison, Katharine Radville, Hayden Kamiya, Emily Zimmerman

**Affiliations:** a Department of Communication Sciences and Disorders, Northeastern University, Boston, MA, USA

## Abstract

Non-nutritive suck (NNS; sucking without feeding) is among the earliest oromotor behaviors in infants and has been shown to be associated with future neurodevelopmental outcomes. Prior work has shown that by 12 months, NNS bursts become shorter and stronger compared to 3 months, but when this shift occurs within the first year remains unclear. Twenty-four full-term infants participated in this prospective, longitudinal study with repeated measures at 3, 6, 9, and 12 months. Infants sucked on a custom pacifier with a pressure transducer for ~5 min. NNS outcomes included burst duration (s), frequency (Hz), amplitude (cmH_2_O), bursts/min, cycles/burst, and cycles/min. As infants aged, their NNS patterns changed significantly. By nine months, the suck duration, bursts/min, cycles/burst, and cycles/min had decreased and by twelve months, these measures showed even further reduction, reflecting continued changes in sucking across the first year of life. The most pronounced NNS changes occurred between 6 and 9 months, coinciding with the introduction of solids and a decreased reliance on NNS. These findings highlight when NNS patterns shift during infancy, providing a reference for identifying age-appropriate targets for infants. This study emphasizes the dynamic nature of NNS during infancy and underscores the need for further assessment and exploration of its developmental trajectory.

## 1. Introduction

**Fig. 1.**
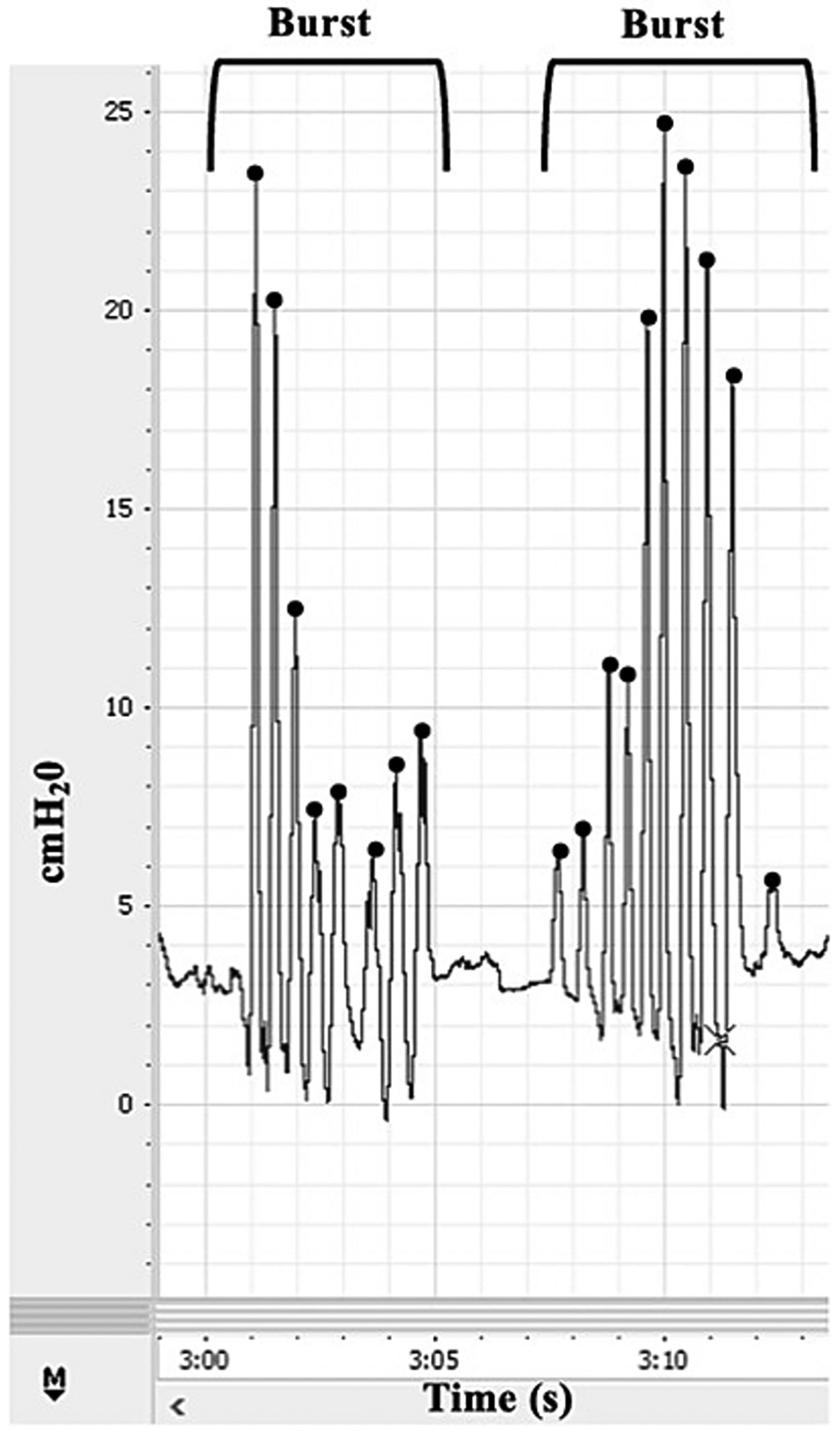
Visual depiction of a stereotypical NNS sample with two bursts.

Non-nutritive suck (NNS; defined as sucking that does not involve feeding) is among the earliest oromotor behaviors observed in infants. NNS appears during fetal development at approximately 15 weeks’ gestational age [1], with organized patterns typically established by 34 weeks’ GA [2]. NNS physiology is characterized by a stereotypical burst–pause pattern, with an intra-burst frequency of approximately 2 Hz and each burst containing 6–12 suck cycles (Fig. 1) [3]. NNS is governed by the suck central pattern generator (sCPG), a bilateral neural network, which projects to lower motor neurons [4,5]. The stability, coordination, and adaptability of NNS rely on intact neural pathways and effective neural integration, making it a sensitive early indicator of central nervous system (CNS) function [6–8].

NNS requires coordinated sucking and breathing, whereas nutritive sucking (NS) also incorporates swallowing, making it a more complex motor task. The suck–swallow–breathe pattern characteristic of NS involves fewer intra-burst pauses, coordinated suction and expression, airway protection, and typically occurs at a slower rhythm of approximately 1 Hz. Thus, the maturation of NNS precedes the development of NS and is an important foundation for the suck–swallow–breathe sequence. Pre-feeding NNS facilitates neural system maturation for rhythmic oral activity [9], resulting in earlier organized sucking emergence [9] and enhanced NS performance [10,11]. While generally used to assist with self-soothing and comfort in full-term infants, use of NNS in neonatal intensive care units can shorten hospital stays, reduce the timeline for reaching full oral feeding competency, and accelerate the transition from tube to oral feeding [12]. NNS performed prior to feeding has also been associated with faster initiation of bottle feeding, improved feeding efficiency, and earlier onset of the first NS burst [13].

Several studies have connected NNS to subsequent neurodevelopmental outcomes. Disruptions in NNS can signal CNS impairment, as evidenced by the high prevalence of sucking and feeding difficulties among infants with neonatal brain injury [8]. Immature or poorly sustained neonatal sucking behaviors have been linked to reduced language, cognitive, and motor functioning in early childhood [14,15]. Notably, existing research has primarily consisted of retrospective studies examining broad neurodevelopmental domains rather than specific outcomes within each domain. Consequently, evidence directly linking NNS to subsequent speech development remains limited. Recent prospective research from our lab on full-term infants has demonstrated a significant associations between longer NNS burst durations, greater cycles per burst, and more cycles per minute at 3 months and lower cognitive and general developmental scores at 12 months [16]. This suggests that infants with more efficient NNS spatiotemportal patterns, characterized by shorter bursts and fewer cycles, demonstrate better developmental outcomes, underscoring the potential value of NNS as a non-invasive, early screening tool for identifying risk for developmental delays.

Prior work has characterized changes in NNS between 3 and 12 months of age in a cohort of 26 full-term infants using a custom pacifier equipped with a pressure transducer to obtain quantitative suck measures [17]. Analyses revealed significant decreases in NNS duration, burst number, cycles per burst, and total cycles from 3 to 12 months and a significant increase in amplitude (suck strength). NNS frequency remained stable, consistent with prior findings that frequency is a less modifiable feature of the sCPG once established [18]. Like changes, we see in nutritive sucking and feeding developmental trajectories, these NNS se differences overtime likely reflect a combination of anatomical growth, neural maturation, and evolving sucking and feeding experiences, including reduced reliance on sucking as solid food intake increases. Clinically, this highlights the importance of understanding age-related changes in NNS to identify deviations from expected trajectories. However, the study only included two timepoints, leaving unanswered questions about *when* during the first year of life the most notable shifts in NNS occur.

More comprehensive longitudinal research with additional timepoints is needed to map the precise developmental trajectory of NNS patterns and identify critical periods of change. The present study expands upon prior work by examining NNS in full-term infants at four timepoints: 3, 6, 9, and 12 months to determine *when* the most substantial changes occur. We hypothesized that a significant shift in NNS, namely a shorter duration with fewer cycles/burst would occur around six months of age as infants are introduced to solid foods and that over time NNS would decrease in duration and increase in strength based on prior findings [17].

## 2. Methods

### 2.1. Participants

Participants were 24 infants who were part of a larger, longitudinal study examining the relation between early sucking and later motor speecch development. Participants were recruited via flyers, social media, and word of mouth. Caregivers consented for their infant to participate, and this study was approved by the institutional review board, IRB #21–08–19, at the authors’ institution. The sample size in this study aligns with prior longitudinal work on NNS that reported statistically significant associations [17]. For the present study, infants were included if they were full-term, had prior pacifier experience. In other words, had used a pacifier at home prior to enrollment in the study because of the influence pacifier experience can have on NNS. Participants included in this smaller study also, had NNS data at 3, 6, 9, and 12 months of age. Infants were excluded if they had any chromosomal or congenital anomalies or diagnosed neurological disorders.

### 2.2. Study timepoints

Prior research has examined the change in NNS between two timepoints, 3 and 12 months of age. The present study expands on this work by including two additional study timepoints 6 and 9 months. The timepoints were chosen as they break the first year of life into 3-month segments. By 3 months of age, families typically have a set home routine or homeostasis. The infant has established their feeding method, still has their suck reflex intact and is primarily relying on sucking skills for nutrition. By six months of age, infants are in or have surpassed the transition feeding phase, a developmental milestone that coincides with significant changes in oral motor function, anatomical growth (including deciduous teeth) and maturation [19,20]. Around 9 months, infants typically show more refined oral motor skills, with emerging chewing movements and improved tongue lateralization. By 12 months of age, most children have several teeth and appropriate CNS timing and coordination to manage cup drinking and more solid foods. These changes are known to influence NNS, which tends to diminish over the first year of life. However, the precise timing and nature of this degradation remain unclear.

### 2.3. Study procedure

Infants participated in study sessions at their homes at 3, 6, and 9 months of age and at our lab at12 months of age. Study visits were purposefully scheduled to occur approximately one hour before the infant’s expected feeding time because other measures (not analyzed for this smaller study) included data collection during bottle feeding. We used a custom-built NNS device that was securely transported in a padded Pelican Case. This portable system features a Philips Avent Soothie pacifier mounted on a handle and connected to an internal pressure transducer. The transducer transmits real-time pressure signals to a PowerLab data acquisition system (ADInstruments, Dunedin, New Zealand), which interfaces with a laptop running LabChart software for data visualization and storage. The same pacifier model and hardware were used consistently across all visits. Detailed information on the device and procedures can be found in [21] [21]. After setting up and calibrating the device, researchers guided caregivers to hold infants in a cradle position and, when possible, presented the pacifier while in a calm and alert state. Each infant used the pacifier for approximately five minutes.

NNS data were processed using LabChart software. Trained coders manually identified suck bursts based on pre-established criteria: each burst consisted of at least two suck cycles with amplitudes exceeding 1 cmH_2_O, separated from other bursts by pauses greater than 1000 milliseconds. These criteria align with prior research on infant NNS [7,22,23]. A custom macro was then used to extract key burst features, including burst duration (s), frequency (Hz), amplitude (cmH_2_O), bursts per minute, cycles per burst, and cycles per minute. For analysis, the two consecutive minutes with the highest number of suck cycles were selected for each infant, and mean values were calculated from that segment.

### 2.4. Statistical analysis

Statistical analyses were performed using GraphPad Prism version 10.3.1 for Mac, GraphPad Software, Boston, Massachusetts USA, www.graphpad.com. A Shapiro-Wilk Test of normal distribution was used to determine normality prior to data analysis. Results of the Shapiro-Wilk Test found that NNS duration, cycles/burst, and cycles/min were not normally distributed. Repeated measures one-way ANOVA (RM ANOVA) was used to compare between timepoints for the normally distributed variables (NNS frequency, amplitude, and bursts/min). The non-parametric version of RM ANOVA, the Friedman test, was used for variables that were not normally distributed (NNS duration, cycles/burst, and cycles/min). When overall effects were significant, post-hoc pairwise comparisons were conducted to examine differences between timepoints. Holm–Šidák correction was applied for normally distributed RM ANOVA variables, and Dunn’s test (Bonferroni-adjusted) was used for Friedman variables. An alpha level of 0.05 was used for all analyses to determine statistical significance.

## 3. Results

Twenty-four full-term infants participated in this study with repeated measures at 3, 6, 9, and 12 months of age.^1^ Infants were 58 % male and, on average, 3.11 months (SD 0.37), 6.02 months (SD 0.24), 8.93 months (SD 0.27), and 12.09 months (SD 0.31) at each respective timepoint. All infants had prior pacifier experience. For full demographics, see Table 1.

**Fig. 2.**
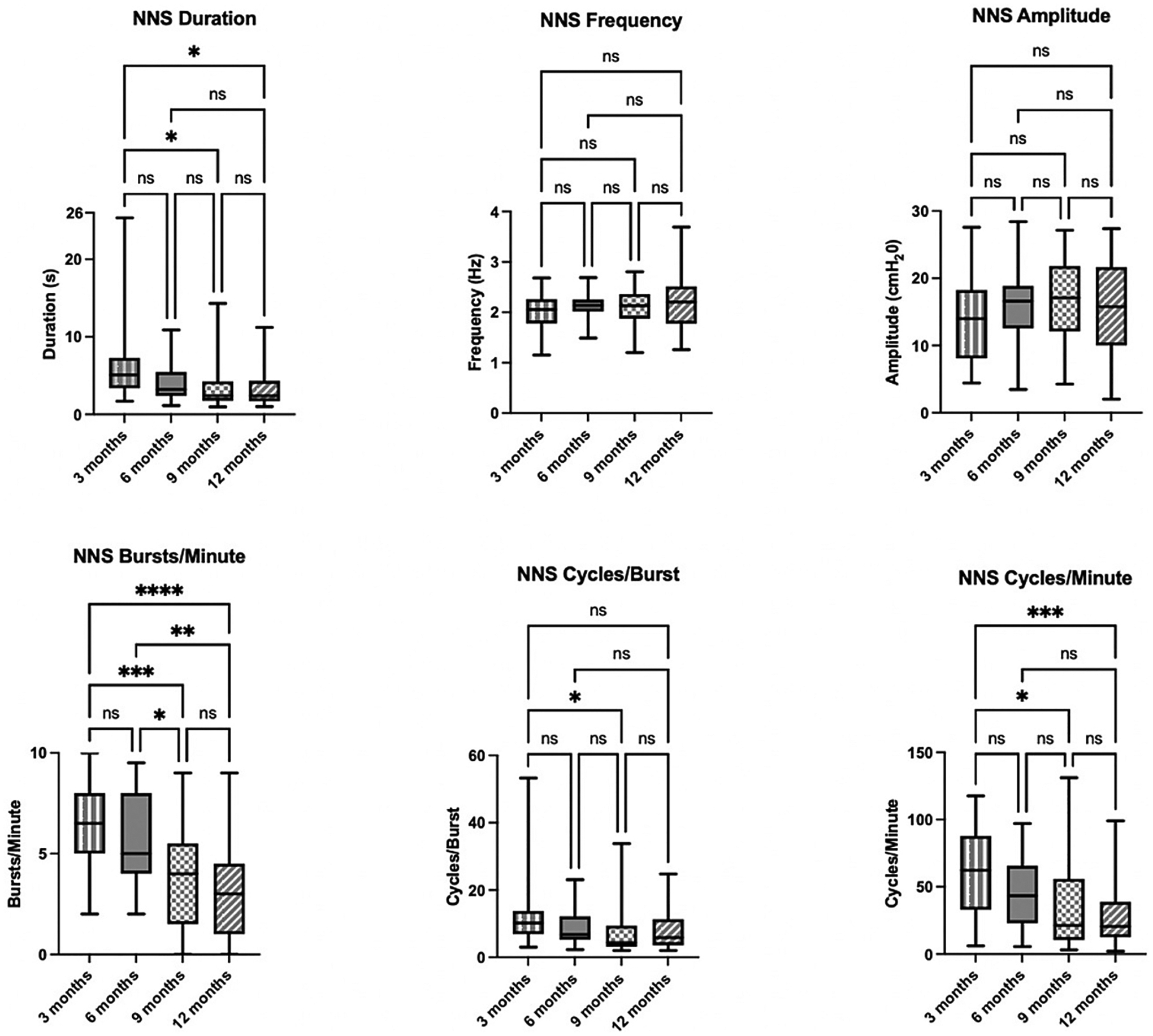
NNS outcomes by age (3-, 6-, 9-, and 12-months)

Statistical analyses showed a significant effect of age on NNS outcomes with the largest difference evident between the 3- and 9- or 3- and 12-month timepoints. NNS duration (*p* = 0.01), bursts/min (*p* = 0.0005), cycles/burst (*p* = 0.02), and cycles/min (*p* = 0.02) significantly decreased between the 3- and 9-month timepoints. Additionally, NNS duration (*p* = 0.01), NNS bursts/min (*p* ≤0.0001), and cycles/min (*p* = 0.0009) significantly decreased between the 3- and 12-month timepoints. Further, NNS bursts/min also decreased between the 6- and 9-month timepoints (*p* = 0.01) and 6- and 12-month timepoints (*p* = 0.002). Taken together, these findings indicate that as infants age, they exhibit a shorter NNS duration with fewer bursts and cycles, see Fig. 2. There were no significant effects of age on NNS frequency and amplitude (suck strength) at any timepoint. Omnibus tests and post-hoc pairwise comparisons for all NNS outcomes are summarized in Table 2.

## 4. Discussion

This study provides the most comprehensive longitudinal characterization of NNS development during the first year of life to date. Consistent with our expectations, results reveal a developmental trajectory with the most pronounced changes occurring between 6 and 9 months of age. These include a reduced NNS burst duration, fewer bursts per minute, and decreased cycles per burst and per minute as infants age. Frequency and amplitude remained stable across timepoints. These results are consistent with and extend beyond previous findings from 3 and 12 months and provide insights into when the most substantial shifts in NNS patterns occur during development.

The identification of 6–9 months as the period of most robust NNS change aligns with several key developmental milestones that converge during this timeframe. This period coincides with the introduction of complementary foods, typically beginning around 6 months, which fundamentally alters an infant’s oral-motor behaviors [19,24]. As infants transition from exclusively liquid nutrition to incorporating solid foods, the functional demands on their oral-motor system shift dramatically [25]. The decreased reliance on sucking for nutrition and soothing may contribute to the observed reduction in NNS parameters during this period.

Neurologically, this timeframe corresponds with significant cortical maturation and the increasing integration of higher-order motor control systems [26,27]. Early NNS patterns are primarily governed by the brainstem-mediated sCPG, which produces the characteristic stereotypical burst-pause patterns observed in younger infants [4]. However, as cortical regions mature and begin to exert greater influence over motor behaviors, the rhythmic, brainstem-driven patterns may give way to more variable, cortically modulated responses [28]. This developmental shift from subcortical to cortical control may explain why we observed preservation of the frequency characteristics (governed by the sCPG) while other parameters showed significant change.

The observed age-related changes in NNS likely reflect multiple converging factors beyond neural maturation. Anatomical growth during this period includes significant changes in oral cavity dimensions, facial structure, and the emergence of deciduous teeth, all of which can influence sucking mechanics [20], and other feeding trajectories. The coordination requirements for NNS may become less challenging as infants develop greater oral-motor control and stability, potentially resulting in more efficient sucking patterns that require fewer cycles and shorter durations to achieve the same functional outcomes.

The stability of NNS frequency across timepoints supports previous research indicating that frequency may be a characteristic of the sCPG that remains relatively invariant once established [17,18]. This suggests that frequency measures may be particularly valuable for clinical assessment, as deviations from expected frequency patterns may signal more significant underlying neural dysfunction than changes in other NNS parameters that naturally vary with development [29].

### 4.1. Clinical implications

Understanding the typical developmental trajectory of NNS has significant implications for clinical practice, particularly in identifying infants who may be at risk for feeding difficulties or broader neurodevelopmental concerns. Our findings indicate that clinicians should consider age-specific norms when evaluating NNS patterns, as what may be considered atypical at 3 months could represent normal development at 9 months. The developmental trajectory illustrated in this study strengthens the evidence base for using NNS as an early screening tool for neurodevelopmental risk. Research has shown that disruptions in typical NNS patterns are associated with later cognitive, language, and motor difficulties [14–16]. This work provides clinicians with a foundation for identifying infants whose NNS patterns deviate significantly from expected patterns, potentially enabling earlier detection of developmental concerns.

The finding that the most substantial changes in NNS patterns occur between 6 and 9 months suggests this period may be particularly valuable for screening purposes. Infants who fail to demonstrate expected developmental changes during this transition may benefit from closer monitoring and earlier intervention services. Infants who maintain prolonged, sustained NNS patterns beyond the typical developmental timeline may be demonstrating persistence of earlier, less mature oral-motor behaviors, which could indicate delayed neural maturation or represent compensatory strategies in response to underlying feeding difficulties.

### 4.2. Limitations and future directions

While this study provides valuable insights into NNS development, several limitations should be acknowledged. The sample included only full-term infants with prior pacifier experience, which may limit generalizability to preterm populations or to full-term infants without pacifier exposure. Future research should examine whether similar developmental patterns occur in preterm infants, accounting for their corrected gestational age. Further, we did not categorize which pacifier brand infants had previously used, and they may have differed from the one used in the study.

Future investigations should also examine the relationship between these normative NNS changes and concurrent developments in other oral-motor behaviors, including nutritive sucking, early speech sound production, and chewing patterns. Understanding how NNS development relates to the broader constellation of oral-motor skills could provide additional insights into the mechanisms driving these developmental changes. Additionally, while we identified 6–9 months as a critical transition period, more frequent sampling within this timeframe could provide greater precision about when these changes begin and how rapidly they occur. It is not yet understood whether the introduction of solid foods drives changes in sucking behavior, or if evolving sucking patterns influence feeding readiness, raising important questions about the directionality and interdependence of these developmental processes.

## 5. Conclusions

This longitudinal study showed that NNS undergoes significant developmental changes during the first year of life, with infants displaying shorter NNS durations, fewer bursts, and fewer cycles with age. These findings provide a more comprehensive description of the development of NNS across the first year of life as compared to what was preivously known. This work adds to growing evidence that early motor behaviors provide valuable insights into neurodevelopmental processes, showing that even basic patterns undergo complex reorganization that reflects the interaction between neural maturation, anatomical growth, and environmental experiences. These findings offer a foundation for translating developmental research into clinical practice and may support the use of NNS assessment as an early screening tool for identifying infants who may require additional support.

## Figures and Tables

**Table 1:** Infant demographics.

	3 months	6 months	9 months	12 months
Age at assessment – Months (SD)	3.11 (0.37)	6.02 (0.24)	8.93 (0.27)	12.09 (0.31)
Sex (% Male)	14 (58 %)			
Birthweight in pounds (SD)	7.46 (0.99)			
Gestational Age (SD)	39.17 (1.11)			
Weight at assessment – Pounds (SD)	12.71 (1.97)	16.40 (2.31)	18.53 (2.36)	20.94 (2.20)

Note: N = 1 participant had missing data for birthweight and gestational age at birth. Additionally, for weight at assessment, *N* = 1 participant had missing data at 3 months, N = 1 at 6 months, N = 3 at 9 months, and *N* = 3 at 12 months.

**Table 2:** Omnibus and Post-hoc Results for NNS Outcomes Across Timepoints.

NNS Variable	Test	Test Statistic (df)	*p* value	Significant post-hoc results
Duration	Friedman	χ^2^(3) = 13.90	0.003	• 3 vs 9 mo: *p* = 0.01 • 3 vs 12 mo: *p* = 0.01
Frequency	RM ANOVA	*F*(2.30, 52.8) = 0.99	0.386	None
Amplitude	RM ANOVA	*F*(2.58, 59.3) = 0.74	0.516	None
Bursts/Min	RM ANOVA	*F*(2.91, 87.4) = 13.26	<0.0001	• 3 vs 9 mo: *p* = 0.0005 • 3 vs 12 mo: *p* < 0.0001 • 6 vs 9 mo: *p* = 0.01 • 6 vs 12 mo: *p* = 0.002
Cycles/Burst	Friedman	χ^2^(3) = 10.68	0.014	• 3 vs 9 mo: *p* = 0.02
Cycles/Min	Friedman	χ^2^(3) = 17.75	<0.001	• 3 vs 9 mo: *p* = 0.02 • 3 vs 12 mo: *p* = 0.0009

## Footnotes

1 Note for NNS bursts/min analysis, seven additional infants had a NNS sample with zero bursts. Therefore, they were included in the bursts/min analysis (total *n* = 31), but excluded from all other analyses because they would not have values for duration, frequency, etc.
